# Genome-Wide Association Study for Ultraviolet-B Resistance in Soybean (*Glycine max* L.)

**DOI:** 10.3390/plants10071335

**Published:** 2021-06-29

**Authors:** Taeklim Lee, Kyung Do Kim, Ji-Min Kim, Ilseob Shin, Jinho Heo, Jiyeong Jung, Juseok Lee, Jung-Kyung Moon, Sungteag Kang

**Affiliations:** 1 Department of Crop Science and Biotechnology, Dankook University, Cheonan 31116, Korea; terry163@gg.go.kr (T.L.); jmkim1206@naver.com (J.-M.K.); sis3761@naver.com (I.S.); heojinho123@kribb.re.kr (J.H.); duddl312@naver.com (J.J.); 2 Seed Management Office, Gyeonggi-do Provincial Government, Yeoju 12668, Korea; 3 Department of Bioscience and Bioinformatics, Myongji University, Yongin 17058, Korea; kyungdokim@mju.ac.kr; 4 Bio-Evaluation Center, Korea Research Institute of Bioscience and Biotechnology, Cheongju 28116, Korea; juseoklee@kribb.re.kr; 5 National Institute of Crop Science, Rural Development Administration, Wanju, Jeonbuk 55365, Korea; moonjk2@korea.kr

**Keywords:** ultraviolet-B, soybean (*Glycine max* (L.) Merrill.), genome-wide association study (GWAS), Axiom^®^ Soya 180K SNP array, DNA repair, photoreactivation, qRT-PCR

## Abstract

The depletion of the stratospheric ozone layer is a major environmental issue and has increased the dosage of ultraviolet-B (UV-B) radiation reaching the Earth’s surface. Organisms are negatively affected by enhanced UV-B radiation, and especially in crop plants this may lead to severe yield losses. Soybean (*Glycine max* L.), a major legume crop, is sensitive to UV-B radiation, and therefore, it is required to breed the UV-B-resistant soybean cultivar. In this study, 688 soybean germplasms were phenotyped for two categories, Damage of Leaf Chlorosis (DLC) and Damage of Leaf Shape (DLS), after supplementary UV-B irradiation for 14 days. About 5% of the germplasms showed strong UV-B resistance, and GCS731 was the most resistant genotype. Their phenotypic distributions showed similar patterns to the normal, suggesting UV-B resistance as a quantitative trait governed by polygenes. A total of 688 soybean germplasms were genotyped using the Axiom^®^ Soya 180K SNP array, and a genome-wide association study (GWAS) was conducted to identify SNPs significantly associated with the two traits, DLC and DLS. Five peaks on chromosomes 2, 6, 10, and 11 were significantly associated with either DLC or DLS, and the five adjacent genes were selected as candidate genes responsible for UV-B resistance. Among those candidate genes, *Glyma.02g017500* and *Glyma.06g103200* encode cryptochrome (*CRY*) and cryptochrome 1 (*CRY1*), respectively, and are known to play a role in DNA repair during photoreactivation. Real-time quantitative RT-PCR (qRT-PCR) results revealed that *CRY1* was expressed significantly higher in the UV-B-resistant soybean compared to the susceptible soybean after 6 h of UV-B irradiation. This study is the first GWAS report on UV-B resistance in soybean, and the results will provide valuable information for breeding UV-B-resistant soybeans in preparation for climate change.

## 1. Introduction

The dosage of ultraviolet (UV) radiation reaching the Earth’s surface has increased since the manufacturing of ozone-depleting gas. Although the production of ozone-depleting substances (ODSs) such as chlorofluorocarbons (CFCs) has been phased out under the 1987 Montreal Protocol, the atmospheric concentration of trichlorofluoromethane (CFC-11) still contributes one-quarter of all chlorine in the atmosphere. Moreover, dichloromethane (CH_2_Cl_2_), an ozone-depleting gas not controlled by the Montreal Protocol, was observed to be increasing rapidly in the atmosphere, becoming another threat to stratospheric ozone [1,2]. These unexpected factors have been delaying a full recovery of the ozone to pre-1970 levels, and the resulting UV light elevation continuously harms life on Earth.

Solar UV radiation is subdivided into three types: UV-A (315–400 nm), UV-B (280–320 nm), and UV-C (200–280 nm). The stratospheric ozone layer absorbs UV-B radiation, but with the depletion caused by ODSs, high levels of UV-B radiation can reach the ground through the ozone hole. Under the enhanced UV-B conditions, plants are negatively affected, with their membranes, proteins, and DNA showing biological and physiological changes associated with photomorphogenesis [3], along with a reduction in biomass accumulation [4]. In plants, the responses to UV-B are related to several biological mechanisms, such as the formation of cyclobutane pyrimidine dimers (CPDs) and pyrimidine-pyrimidone (6-4) photoproducts [5,6], inactivation of photosynthesis [7], downregulation of phytohormones [8], secondary metabolism [9], and free-radical scavenging [10]. CPD, the most abundant DNA damage induced by UV-B, inhibits transcription and replication, leading to mutagenesis in plants [11,12]. The UV-B-induced CPDs are repaired by mechanisms including nucleotide excision repair (NER) and photoreactivation [13,14,15]. The other mechanism can be activated by UV RESISTANCE LOCUS8 (UVR8) homodimers [16]. This UV-B-specific photoreceptor interacts with CONSTITUTIVELY PHOTOMORPHOGENIC1 (COP1) and regulates the gene expression response to UV-B acclimation. Recent studies revealed that UV-induced responses such as changes in phytohormone and metabolite levels also depend on UVR8 [8,17,18].

Soybean is considered a UV-B-sensitive plant. Enhanced UV-B turns the soybean plants into dwarf type by shortening the internode length [19]. Feng, An [20] indicated that soybean under UV-B enhanced conditions showed changes in flowering time and decreases in chlorophyll a/b contents, total leaf number, and total leaf area. Moreover, decreases in total biomass and yield components such as seed size and weight and reductions in concentrations of phenolic compounds and isoflavones were observed in soybean plants when exposed to elevated UV-B radiation [7,21,22]. Recent studies in soybean have investigated the underlying genes and variations controlling these responses to UV-B light. Several quantitative trait loci (QTLs) associated with UV-B resistance were identified using the F_11_ recombinant inbred line (RIL) population of Keunol (UV-B susceptible) × Iksan 10 (UV-B-resistant; derived from Bangsa by irradiation breeding) [23]. In a study with the F_12_ RIL population of Keunol × Iksan 10, positional mapping of a QTL on chromosome 7 identified *RAD23*, a homolog of yeast *RAD23*, as one of the candidate genes for UV-B resistance [24]. In yeast, RAD23 was previously reported as a UV excision repair protein, suggesting a similar function regarding UV-B resistance in soybean. In another study, a genome-wide comparison between UV-B-resistant IT162669 and UV-B-sensitive Cheongja 3 identified four genes related to plant protection including UV-B resistance [25]. Furthermore, using an F_6_ RIL population of Cheongja 3 × Buseok, four UV-B resistance QTLs were identified, and among those QTLs, a gene with two non-synonymous SNPs differentiating the parental lines, was identified on chromosome 6 [26].

Genome-wide association studies (GWASs) are a powerful approach that provides higher resolution than linkage mapping because they utilize the historical recombination events of a natural population [27]. Numerous GWASs have been conducted in soybean to identify putative genes and QTLs associated with agronomic traits such as branching [28], plant height [29], photosynthesis [30], resistance to insect pests [31], and soybean mosaic virus [32]. This was made possible by the availability of high-throughput SNP genotyping systems such as Golden Gate assay [33], SoySNP50K array [34], and Axiom^®^ Soya 180K SNP array [35]. Nevertheless, for UV-B resistance genes and QTLs, a GWAS has not yet been conducted on a soybean germplasm collection. In this study, we performed a GWAS for a total of 688 soybean germplasms and identified eight genes associated with UV-B resistance. Furthermore, we compared the expression level of two candidate genes using real-time quantitative RT-PCR (qRT-PCR) between UV-B-resistant (GCS731) and susceptible (Daepung [35]) genotypes, showing significantly higher expressions in the UV-B-resistant genotype.

## 2. Results

### 2.1. Phenotypic Evaluation of Soybean Germplasm Collection under Enhanced UV-B Conditions

**Figure 1:**
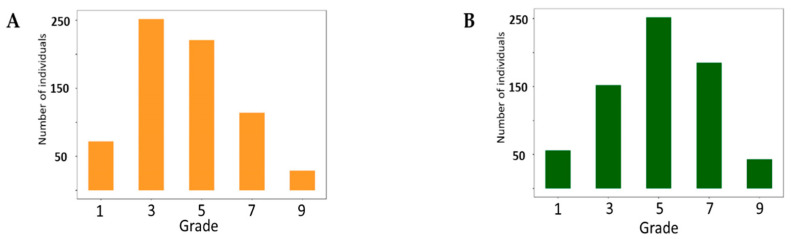
Phenotypic distributions of 688 soybean germplasm responses to enhanced UV-B radiation. (**A**) Damage of Leaf Chlorosis (**B**) Damage of Leaf Shape. Grade 1: same degree of damage with resistant soybean (GCS731). Grade 7: same degree of damage with susceptible soybean (Daepung).

A total of 688 soybean germplasms were evaluated under UV-B enhanced conditions for two different phenotypic change responses to UV-B light: Damage of Leaf Chlorosis (DLC) and Damage of Leaf Shape (DLS) (Table S1). The phenotypic distribution of DLS showed a similar pattern to the normal distribution (Figure 1). Seventy-two (10.5%) and 59 (8.6%) accessions were scored as grade 1 for DLC and DLS, respectively, and 36 (5.2%) accessions were scored as grade 1 for both DLC and DLS. Among these 36 accessions, GCS731 (IT025231) showed the most resistant phenotype.

### 2.2. Genome-Wide Association Study (GWAS)

**Figure 2:**
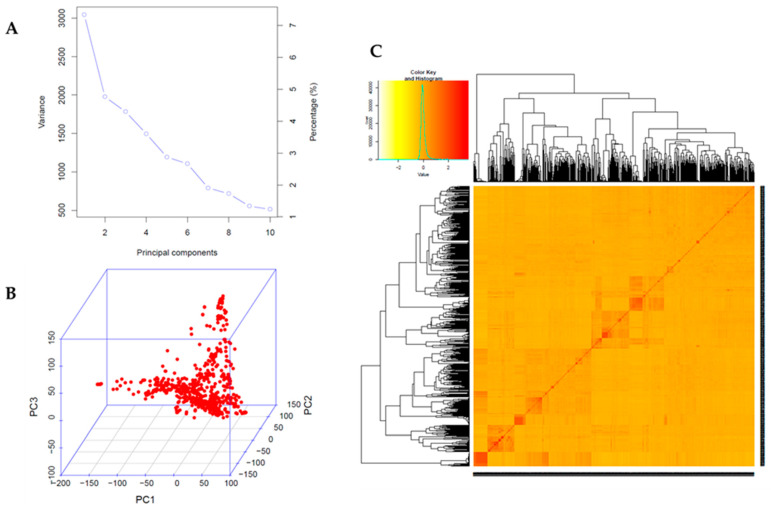
Population structure analysis of 688 soybean germplasm collection. (**A**) Variance of first 10 principal components reflected by 65,762 SNPs used in the GWAS. (**B**) Principal component analysis (PCA) of 65,762 SNPs indicating the population structure of 688 soybean accessions. (**C**) A heatmap of kinship matrix presenting genetic relatedness of 688 soybean accessions.

**Figure 3:**
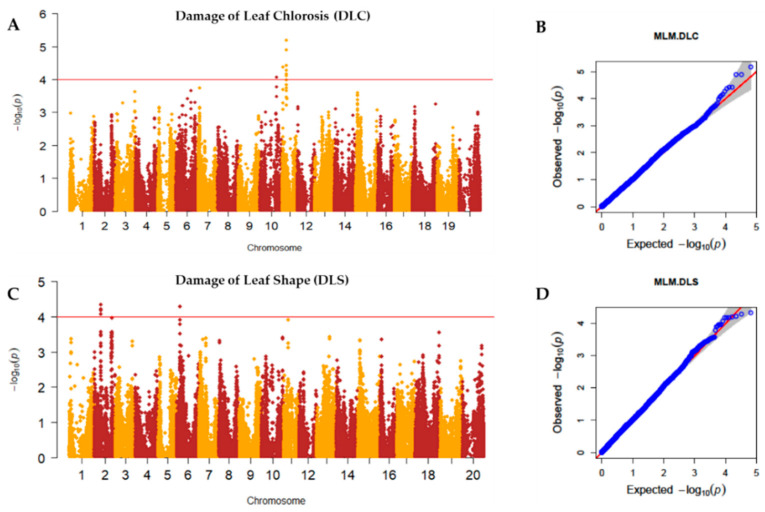
Manhattan plots and quantile–quantile (QQ) plots of UV-B resistance traits. The upper red lines of the Manhattan plots indicate the threshold (-log(*p*) = 4) for significant SNPs (FDR-unadjusted *p*-value < 0.0001) (**A**) Manhattan plot for DLC (Damage of Leaf Chlorosis); (**B**) QQ plot for DLC; (**C**) Manhattan plot for DLS (Damage of Leaf Shape); (**D**) QQ plot for DLS.

Principal component analysis (PCA) and kinship analyses were conducted for population structure analysis using 65,762 high-quality SNPs with a compressed mixed linear model (CMLM). The variation of the first 10 principal components (PCs) showed an inflection point at PC2, suggesting that the first two PCs dominated the population structure on the association mapping (Figure 2A,B). Weak genetic relatedness of the population was observed from the distribution of the coefficients from kinship analysis among the 688 germplasms (Figure 2C). To identify SNPs associated with the UV-B-induced traits DLC and DLS, a GWAS was conducted for the 688 soybean germplasms using GAPIT [36]. The observed *p*-values of MLM followed the expected *p*-values of that for both DLC and DLS as shown in quantile–quantile plots (QQ plots), indicating that there is little chance of false positives due to the population structure (Figure 3). A total of five peaks were significantly associated with DLC and DLS with a *p*-value (FDR-unadjusted) threshold of 0.0001 (Table S2). No SNP was found at FDR-adjusted *p*-value < 0.05 due to high stringency [37]. For association with DLC, a single peak on chromosome 10 and two peaks on chromosome 11 were identified. AX-90454793 on chromosome 10 and AX-90522955 and AX-90521132 on chromosome 11 were significantly associated with DLC with -log(*p*) = 4.06, 4.38 and 5.19, respectively. For association with DLS, single peaks were identified on both chromosomes 2 and 6. AX-90334094 on chromosome 2 and AX-90333167 on chromosome 6 were significantly associated with DLS with -log(*p*) = 4.34 and 4.29, respectively. In total, five SNPs (chromosomes 2, 6, 10, and 11) were significantly associated with UV-B resistance traits, DLC and DLS (Table S2).

### 2.3. Linkage Disequilibrium (LD) Analysis

**Figure 4:**
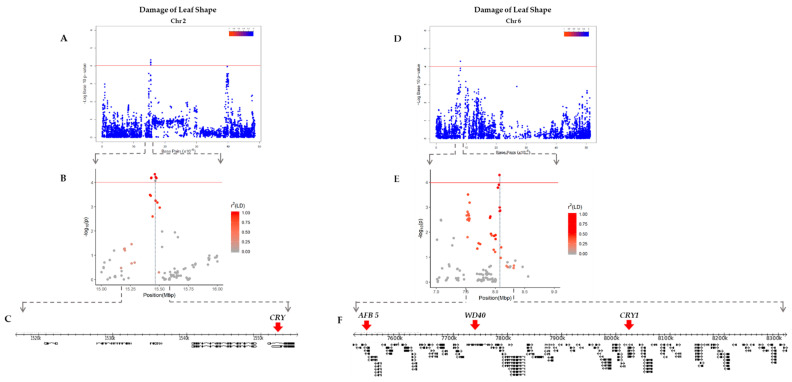
Identification of candidate genes associated with DLS (Damage of Leaf Shape) on chromosomes 2 and 6. (**A**) Manhattan plot of chromosome 2 with the threshold (-log(*p*) = 4) for significant SNPs. (**B**) Scatter plot centered on AX-90334094 with r^2^ (LD) values. (**C**) Gene models presented within the LD block region. The candidate genes for UV-B resistance are marked with red arrows. (**D**) Manhattan plot of chromosome 6 with the threshold (-log(*p*) = 4) for significant SNPs. (**E**) Scatter plot centered on AX-90333167 with r^2^(LD) values. (**F**) Gene models present within the LD block region. The candidate genes for UV-B resistance are marked with red arrows.

The ranges of LD blocks that contain significant SNPs were estimated to narrow down the candidate genes nearby. For DLC, three peaks on chromosomes 10 and 11 represented by AX-90454793, AX-90522955, and AX-90521132 were examined (Table 1). AX-90454793 on chromosome 10 was found inside an LD block 292 bp in size with a gene not associated with UV-B resistance (Figure S1). A 19,194 bp-long LD block carrying AX-90522955 on chromosome 11 also contained 18 genes not related to UV-B resistance (Figure S2), while AX-90521132 was located within another LD block on chromosome 11 that was 24,174 bp in length with eight genes including *Glyma.11g130800*, which encodes the WD40 domain (Figure S3). For DLS, LD blocks comprising the peaks on chromosomes 2 and 6 were determined. AX-90334094 on chromosome 2 was located inside an LD block spanning 29,095 bp with nine genes (Figure 4A–C), while the LD block on chromosome 6 carrying AX-90333167 was 798,909 bp in size with 94 genes including *Glyma.06g103200*, which encodes CRY1 (Figure 4D–F).

For each gene within these LD blocks, gene descriptions suggesting their potential functions were obtained from SoyBase and carefully curated to select genes that have functions related to UV-B resistance. We selected a total of five putative genes related to UV-B perception and resistance mechanisms such as WD40 domain, auxin-related protein, and photolyase (Table 2). Most interestingly, *Glyma.02g017500* and *Glyma.06g103200* encoding cryptochromes are reported to have a similar structure to photolyase, which plays a role in repairing UV-B damaged DNA by photoreactivation [38].

### 2.4. Gene Expression Analysis by qRT-PCR

**Figure 5:**
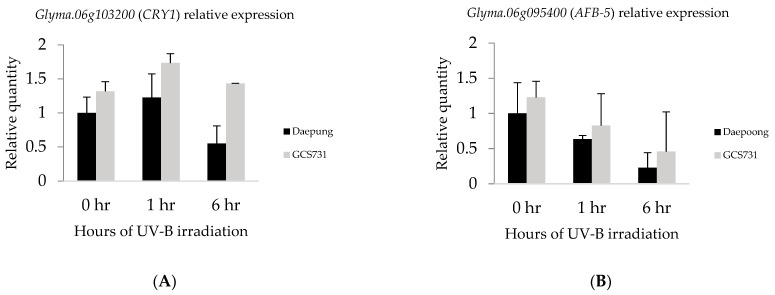
Comparisons of gene expression levels between susceptible “Daepung” and resistant “GCS731” under UV-B irradiation of 0, 1, and 6 h. (**A**) Relative expression levels of *Glyma.06g103200* encoding cryptochrome (CRY1). (**B**) Relative expression levels of *Glyma.06g095400* encoding Auxin F-box 5 (AFB5).

Expression levels of the candidate genes were measured using qRT-PCR to identify UV-B-induced changes in gene expression under UV-B enhanced conditions. UV-B-resistant (GCS731) and susceptible (Daepung) soybean plants were treated with supplementary UV-B radiation for three different durations (0, 1, and 6 h) with three replicates. Total RNA was isolated from trifoliate leaves of the two genotypes, and qRT-PCR was conducted for two candidate genes, *Glyma.06g103200* (*CRY1*) and *Glyma.06g095400* (*AFB5*). For both genes, the expression levels were higher in UV-B-resistant GCS731 than UV-B susceptible Daepung throughout all three UV-B conditions (Figure 5A,B). Gly-ma.06g103200 (CRY1) was expressed more after being exposed to enhanced UV-B radiation for 1 h, and then its expression decreased at 6 h of irradiation for both resistant and susceptible genotypes (Figure 5A). Interestingly, a statistically significant and nearly three-fold difference was observed between GCS731 and Daepung after 6 h of UV-B irradiation (Figure 5A). On the other hand, the expression levels of *Glyma.06g095400* (*AFB5*) remained downregulated when exposed to supplementary UV-B radiation regardless of the duration (Figure 5B). However, the difference between the two genotypes was not statistically significant.

## 3. Discussion

In this study, a total of 688 soybean germplasms from diverse genetic backgrounds were evaluated for their responses to enhanced UV-B radiation. Evaluated by two distinct phenotypic criteria based on changes in leaf chlorosis (DLC) and shape (DLS), we identified 36 accessions (5.2% of total germplasms) showing strong UV-B resistance with DLC and DLS scores of 1 (most resistant). For both DLC and DLS, the distributions of phenotype scores were close to a normal distribution and showed transgressive segregation. These results indicate that DLC and DLS are quantitative traits governed by polygenes. The plant has many direct and indirect effects from UV-B radiation such as damage to DNA, proteins, and membranes [39]. Thus, resistance to UV-B radiation is strongly related to various mechanisms involving DNA repair, perception of UV-B radiation, and removal of oxidative stress.

Based on the GWAS results, we selected a total of five genes involved in response mechanisms to UV-B radiation. Among these genes, *Glyma.11g130800* and *Glyma.06g097800* encode the WD40 domain, which plays an essential role in UV-B resistance. The WD40 domain is one of the most abundant domains that function as platforms for protein–protein interactions and are involved in numerous biological processes including DNA damage and repair [40]. COP1 carries the WD40 domain and interacts with UVR8, the specific UV-B receptor. Moreover, RUP1 and RUP2 are members of the WD40 repeat protein family and interrupt the interaction between UVR8-COP1 [41]. Interestingly, many protein-containing “WDxR” motifs in their WD40 repeat regions enhance the repair of damaged DNAs as seen in Cockayne syndrome A (CSA) [42], damaged DNA binding protein 1 (DDB1) [43], and WRAP53 beta [44].

Auxin is a plant hormone that promotes cell elongation. Plants may bend their shoots and roots in response to UV-B radiation by managing plant hormones [45,46], and several studies have reported that UV-B radiation inhibits auxin biosynthesis [17,47]. *Glyma.06g095400* encodes Auxin F-box protein 5 (AFB5), and AFB5 is known as an auxin receptor that plays a role in mediating the leaf cell expansion and division by auxin [48,49]. The regulation of auxin is linked to the transcription factors central to UV-B signaling. For example, HY5, a transcription factor of auxin signaling and transportation, is an essential factor for UV-B perception signaling [46,50,51]. Therefore, *Glyma.06g095400* might be involved in UV-B signaling regulating auxin in soybean.

*Glyma.02g017500* and *Glyma.06g103200* encode cryptochrome (CRY) and cryptochrome 1 (CRY1) in soybean, respectively. Cryptochrome is a flavoprotein photoreceptor that senses blue light to regulate plant development and circadian clock [52]. The structure of cryptochrome is similar to that of the photoreactivation enzyme, which is considered an ancient photolyase [38]. Photolyase is also a flavoprotein that has a role in eliminating UV-induced CPDs in DNA during photoreactivation [13,38,52]. Photoreactivation was described in 1949 as a DNA repair mechanism through which photodimers are removed by binding of photolyase to the damaged region [53]. Although cryptochrome is considered a protein that reduces DNA repair activity and has a novel function in signaling, several studies have shown that cryptochrome still acts as a DNA repair enzyme [54]. DNA repair is well known as a key process to overcome UV-induced damage. Thus, *Glyma.02g017500* and *Glyma.06g103200* could be important enzymes that promote UV-B resistance in soybean. Similarly, an orthologous gene of *RAD23* reported as an NER-related gene has been reported as a gene associated with UV resistance in soybean [24].

For *Glyma.06g103200* (*CRY1*) and *Glyma.06g095400* (*AFB5*), the gene expression levels between UV-B-resistant and susceptible soybeans were compared using qRT-PCR. *CRY1* showed higher expression in a resistant soybean (GCS731) throughout the different irradiation times. In addition, the expression levels after 1 and 6 h of UV-B irradiation showed significant differences between the two genotypes, suggesting an induced expression of *CRY1* in the resistant genotype. Furthermore, Auxin F-box 5 (AFB5) in both soybean varieties showed further downregulation as UV-B was irradiated for a longer time. In the resistant genotype, AFB5 was expressed higher compared to the susceptible genotype; however, the difference was not statistically significant. This might be because the auxin hormone is commonly spread out in plants for growth and development as well as UV-induced response [48].

In this study, we selected genetic resources, including GCS731, which showed the strongest UV-B resistance among 688 soybean germplasms. These resources can be incorporated into a breeding program to develop soybean varieties resistant to enhanced UV-B radiation. Due to global warming, crop cultivation may expand to higher latitude to avoid pests and diseases that become more severe in warm environments. Moreover, warmer temperature stimulates the depletion of the ozone layer, resulting in elevated UV-B radiation throughout crop cultivation. Therefore, UV-B-resistant varieties can be cultivated at higher latitude under elevated UV-B radiation without any loss of production.

As most plants produce energy from solar radiation, they evolved to tolerate UV-B radiation throughout the entire life cycle. However, not much has been reported about UV-B resistance in soybean, while numerous studies have covered various aspects of resistance mechanisms in the model plant *Arabidopsis*. This study is the first to report a GWAS analysis conducted with a large set of soybean germplasms. We identified five significant peaks covering five genes with their potential functions related to UV-B responses. Further investigation of these candidate genes of UV-B resistance would give us insights into how the resistant soybean responds to enhanced UV-B radiation. Furthermore, evaluating soybean transplants overexpressing the candidate genes would validate the exact function of these genes under high intensity of UV-B radiation.

## 4. Materials and Methods

### 4.1. Plant Materials and Growth Conditions

A collection of a total of 688 soybean germplasms consisting of 620 landraces, 33 breeding lines, 29 varieties, and 6 accessions with unknown origin were obtained from the National Institute of Crop Science in the Rural Development Administration (RDA, Wanju, Korea) (Table S1). In the collection, 620 accessions were from South Korea, and 12, 9, 4, and 1 accessions were from the USA, Japan, China, and North Korea, respectively. Soybean plants were cultivated in a growth chamber and an experimental greenhouse at Dankook University, Cheonan, Korea. With Daepung [35] (UV-B susceptible) and GCS731 (UV-B-resistant) as control plants, each accession was planted in 27 cm wide × 53 cm long trays with 50 holes filled with synthetic cultivation soil and organized in a completely randomized design with three replicates.

### 4.2. Evaluation of UV-B Resistance

**Figure 6:**
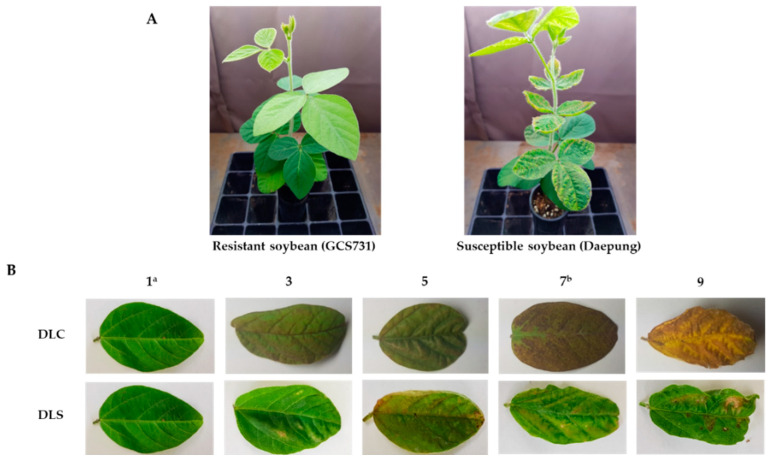
(**A**) Phenotypic differences between resistant soybean (GCS731) and susceptible soybean (Daepung) after 2 weeks of UV-B irradiation. (**B**) Levels of leaf damage after 2 weeks of UV-B irradiation in soybean. 1: 0–10% damage, 3: 10–30% damage, 5: 30–50% damage, 7: 50–70% damage, 9: 70–90% damage, DLC: Damage of Leaf Chlorosis, DLS: Damage of Leaf Shape. 1^a^: same degree of damage with resistant soybean (GCS731). 7^b^: same degree of damage with susceptible soybean (Daepung).

Evaluation of UV-B resistance under enhanced UV-B radiation was conducted according to a previous study with minor modifications [23,24]. Artificial irradiation systems using UV-B lamps (TL 20W/01 RS 312 nm UV-B narrowband lamp, Philips) were installed in the growth chamber and the greenhouse at Dankook University, Cheonan, Korea. The soybean plants were transferred to the UV-B irradiation system and irradiated at the V2 stage for 6 h per day (10:00–16:00) for 14 days. For uniform UV-B intensity of 3.0–3.5 Wm^−2^, the distances from the UV-B lamps to the tops of the plants were maintained between 20 and 30 cm [24]. The UV-B intensity was checked repeatedly using a UV-radiometer (DO 9847, Delta OHM) with an LP 471 UV-B sensor. After 14 days of irradiation, the levels of leaf damage due to supplemental UV-B light were measured. The degree of leaf chlorosis (DLC) and the degree of leaf shape change (DLS) were determined with scores from 1 to 9 by comparing the phenotypes with control plants within each tray (“1” for 0–10% damage, “3” for 10–30% damage, “5” for 30–50% damage, “7” for 50–70% damage, and “9” for 70–90% damage) (Figure 6). To minimize phenotyping errors, soybean accessions with each measurement below three on average (DLC < 3 or DLS < 3) were tested twice with three replicates per accession.

### 4.3. Genome-Wide Association Study (GWAS)

For population structure analysis, PCA and kinship plots were generated using the genome association and prediction integrated tool (GAPIT) package in the R program [55] and a heat map of kinship matrix was created using the VanRaden kinship algorithm [56]. SNP genotyping data of soybean germplasms generated using 180K Axiom^®^ Soya SNP array [35] were obtained from the National Institute of Crop Science in the Rural Development Administration (RDA, Wanju, Korea). SNPs with a minor allele frequency under 5% (MAF < 5%) were excluded to remove low-quality SNPs, which generated a final set of 65,762 high-quality SNPs for GWAS analysis. The GWAS was performed using GAPIT [55] with default settings. A mixed linear model (MLM), which generally outperforms a general linear model (GLM) by adding random effects of SNPs, was used to analyze associations between SNP genotypes and UV-B responses. The false discovery rate (FDR) adjusted *p*-values from GAPIT were found to be very stringent because the marker effects could have been overcorrecting based on both population structure Q and kinship K [57,58]. Therefore, an FDR-unadjusted *p*-value of 0.0001 was used as the threshold to identify significant SNPs for UV-B resistance.

### 4.4. Linkage Disequilibrium (LD) Analysis and Candidate Gene Identification

LD analysis was performed using PLINK software with an LD window length of 1 Mb and an unlimited number of variants within the LD window (-r2-ld-window-kb 1000 –ld-window 99999) [28,59]. LD blocks containing significant SNPs were searched using the genome browser at Phytozome (https://phytozome.jgi.doe.gov/jbrowse/, accessed on 21 June 2021) to narrow down candidate genes responsible for UV-B resistance. Functional descriptions of candidate genes were obtained from the SoyBase website (https://soybase.org/gb2/gbrowse/, accessed on 21 June 2021) [60].

### 4.5. Real-Time Quantitative Reverse Transcription PCR

For RNA extraction, a resistance soybean GCS731 and a susceptible soybean Daepung were irradiated by UV-B at the V2 stage with three replicates. Fully expanded trifoliate leaves were harvested from each plant according to the irradiation time: 0, 1, 6 h [61]. The harvested leaves were frozen immediately in liquid nitrogen and ground into a powder with beads in a 2 mL tube. Total RNA was isolated from the ground leaves using the RNeasy Mini Kit (QIAGEN). The quantity of total RNA was estimated using Nanodrop, and 1 μg of total RNA was used for reverse transcription. First-strand cDNA was synthesized using cDNA EcoDry Premix (TaKaRa, Clontech, Cellartis).

Real-time quantitative reverse transcription PCR (qRT-PCR) was performed on an ABI 7500 real-time PCR machine with 2× QuantiTect SYBR Green PCR Master Mix (Biosystems, Germantown, MD, USA). The primers of genes were designed based on CDS sequences obtained at Phytozome. To minimize amplification error due to DNA contamination, primer sets were designed from exon–exon junctions. The amount of cDNA was normalized to the β-ACTIN reference gene [62]. Two replicate reactions were performed for each sample, and the 2^−ΔΔCT^ method [63] was used to convert the Ct value (quantification cycles) into the relative quantities. The amplification conditions were as follows: 95 °C for 5 min followed by 40 cycles of 95 °C for 5 s, 60 °C for 10 s, and 95 °C for 15 s. The melting temperature was 60 °C for 1 min.

## 5. Conclusions

In this study, we identified UV-B-resistant soybeans by evaluating the phenotypic changes of 688 soybean germplasms under UV-B enhanced conditions. We conducted a GWAS analysis for UV-B resistance, which is first to report on soybean, resulting in five significant peaks that narrowed into five candidate genes related to UV-B responses. Among these genes, *CRY* and CRY1 were the most presumed genes for UV-B resistance due to their function associated with the DNA repair process. Cryptochrome protein is structurally similar to photolyase, which repairs damaged DNA in a photoreactivation process. Furthermore, the expression level of *CRY1* was statistically significantly higher in resistance soybean GCS731 compared to susceptible soybean Daepung. SNP markers AX-90334094 and AX-90333167 with the candidate genes *CRY* and *CRY1* could be used in marker-assisted selection when breeding UV-B-resistant soybean.

## Figures and Tables

**Table 1:** List of single nucleotide polymorphisms significantly (FDR unadjusted *p*-value < 0.0001 or -log(*p*) > 4) associated with DLC (Damage of Leaf Chlorosis) and DLS (Damage of Leaf Shape).

Trait	Chr.	SNP	Position	FDR Unadjusted *p*-Values	−log(*p*)	maf	R^2^ without SNP	R^2^ with SNP	FDR Adjusted *p*-Values	Effect	Linkage Disequilibrium Block	Distance	No. of Genes
DLC	10	AX-90454793	38322497	8.65 × 10^−5^	4.06	0.16	0.02	0.04	0.57	−0.46	382,934~383,225	292 bp	1
	11	AX-90522955	1168027	4.22 × 10^−5^	4.38	0.40	0.02	0.05	0.46	0.37	9,939,957~1,013,188	19,194 bp	18
		AX-90521132	9939957	6.50 × 10^−6^	5.19	0.08	0.02	0.05	0.28	−0.69	1,144,379-1,168,553	24,174 bp	8
		AX-90466824	9955814	7.83 × 10^−5^	4.11	0.16	0.02	0.05	0.57	−0.45	-	-	-
		AX-90325683	10099676	1.27 × 10^−5^	4.90	0.08	0.02	0.05	0.28	−0.65	-	-	-
		AX-90318887	10104602	1.27 × 10^−5^	4.90	0.08	0.02	0.05	0.28	−0.65	-	-	-
		AX-90379400	10109488	3.77 × 10^−5^	4.42	0.10	0.02	0.05	0.46	−0.57	-	-	-
		AX-90353742	10119213	3.73 × 10^−5^	4.43	0.10	0.02	0.05	0.46	−0.58	-	-	-
		AX-90477047	10121857	6.84 × 10^−5^	4.16	0.10	0.02	0.05	0.56	−0.56	-	-	-
		AX-90427669	10126101	5.30 × 10^−5^	4.28	0.10	0.02	0.05	0.50	−0.58	-	-	-
DLS	2	AX-90319706	15429030	6.67 × 10^−5^	4.18	0.18	0.05	0.07	0.63	−0.45	-	-	-
		AX-90457128	15430402	6.26 × 10^−5^	4.20	0.18	0.05	0.08	0.63	−0.46	-	-	-
		AX-90334094	15460775	4.56 × 10^−5^	4.34	0.18	0.05	0.08	0.63	−0.47	1,516,984~1,550,224	29,095 bp	9
		AX-90344232	15464333	0.0000847	4.07	0.19	0.05	0.07	0.64	−0.45	-	-	-
		AX-90436068	15467717	6.09 × 10^−5^	4.22	0.18	0.05	0.08	0.63	−0.46	-	-	-
		AX-90474824	15473795	6.60 × 10^−5^	4.18	0.18	0.05	0.07	0.63	−0.45	-	-	-
		AX-90329251	15474547	6.60 × 10^−5^	4.18	0.18	0.05	0.07	0.63	−0.45	-	-	-
	6	AX-90333167	8071856	5.13 × 10^−5^	4.29	0.23	0.05	0.08	0.63	−0.43	7,516,555~8,315,464	798,909 bp	94

**Table 2:** Information of candidate genes for UV-B resistance.

Trait	Chr.	Linkage Disequilibrium Block	Candidate Gene	Gene Position	Description
DLC ^1^	11	9,939,957–10,131,882	*Glyma.11g130800*	9,983,698–9,993,001	Transducin/WD40 repeat-like superfamily protein, WD40 repeat family
DLS ^2^	2	1,516,984–1,550,224	*Glyma.02g017500*	1,550,874–1,554,518	CRYPTOCHROME, photolyase/blue-light receptor 2, DNA repair, DNA photolyase activity
	6	7,516,555–8,315,464	*Glyma.06g095400*	7,530,783–7,533,737	Auxin F-box protein 5, protein binding
			*Glyma.06g097800*	7,725,465–7,766,431	Protein binding, related BEACH and WD40 repeat proteins, WD domain
			*Glyma.06g103200*	8,205,073–8,210,842	CRYPTOCHROME 1, DNA repair, blue/ultraviolet sensing protein C terminal, DNA photolyase activity

^1^ DLC: Damage of Leaf Chlorosis. ^2^ DLS: Damage of Leaf Shape.

## Data Availability

Not applicable.

## Supplementary Materials

The following are available online at https://www.mdpi.com/article/10.3390/plants10071335/s1, Figure S1: Identification of the candidate genes associated with DLC (Damage of Leaf Chlorosis) on chromosome 10, Figure S2: Identification of the candidate genes associated with DLC (Damage of Leaf Chlorosis) on chromosome 11, Figure S3: Identification of the candidate genes associated with DLC (Damage of Leaf Chlorosis) on chromosome 11, Table S1: Information of 688 soybean germplasms used in this study.
