# Supplementary material for: Genome-Wide Association Study for Ultraviolet-B Resistance in Soybean (Glycine max L.)
Source: Plants (Basel). 2021 Jun 29;10(7):1335. doi: 10.3390/plants10071335 (PMC8308986; doi:10.3390/plants10071335)
Supplement: Supplementary file 1 [file plants-10-01335-s001.zip › supplemental figure_210622.pptx]

## Slide 1
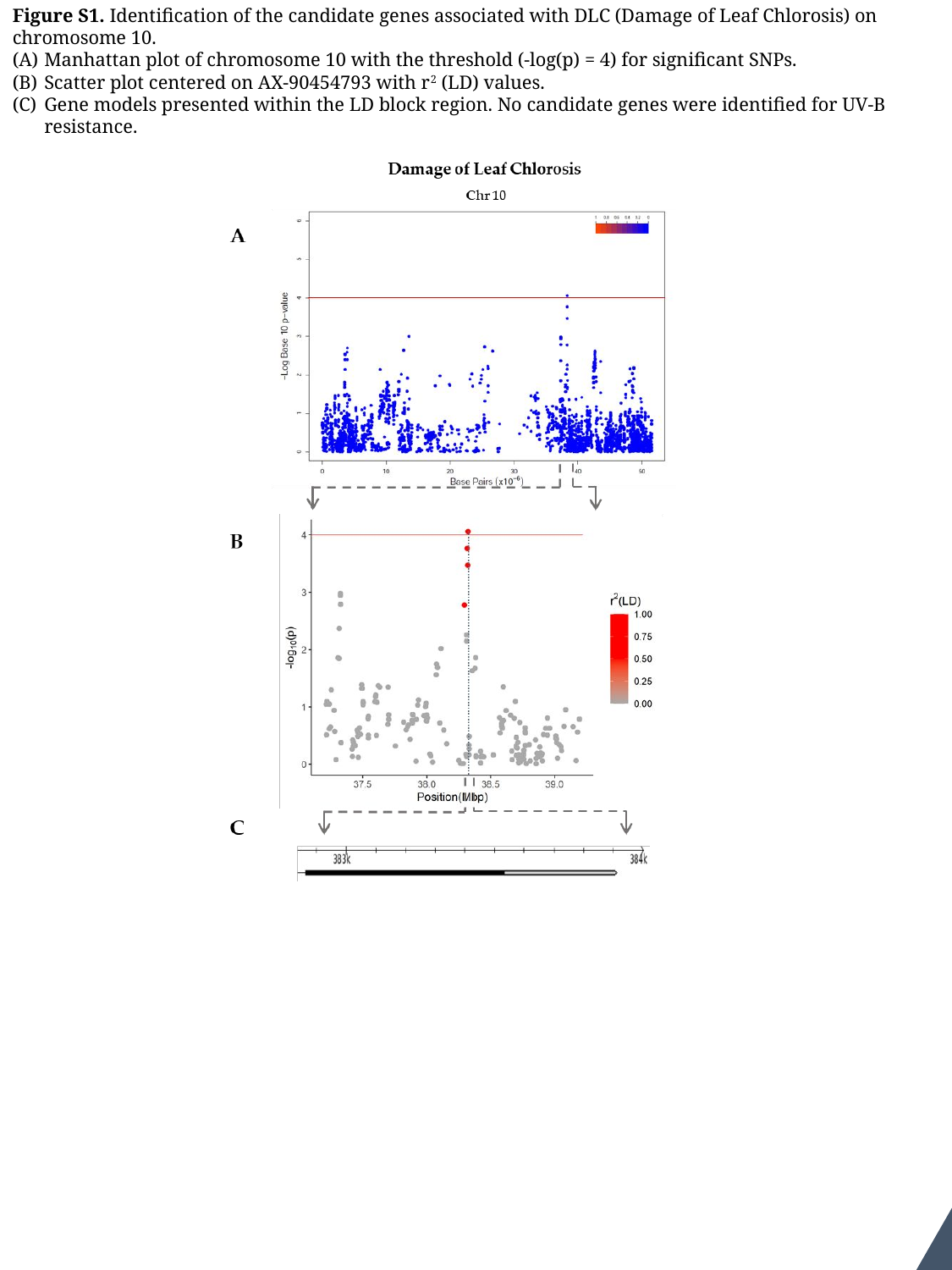

Figure S1. Identification of the candidate genes associated with DLC (Damage of Leaf Chlorosis) on chromosome 10.
Manhattan plot of chromosome 10 with the threshold (-log(p) = 4) for significant SNPs.
Scatter plot centered on AX-90454793 with r2 (LD) values.
Gene models presented within the LD block region. No candidate genes were identified for UV-B resistance.

## Slide 2
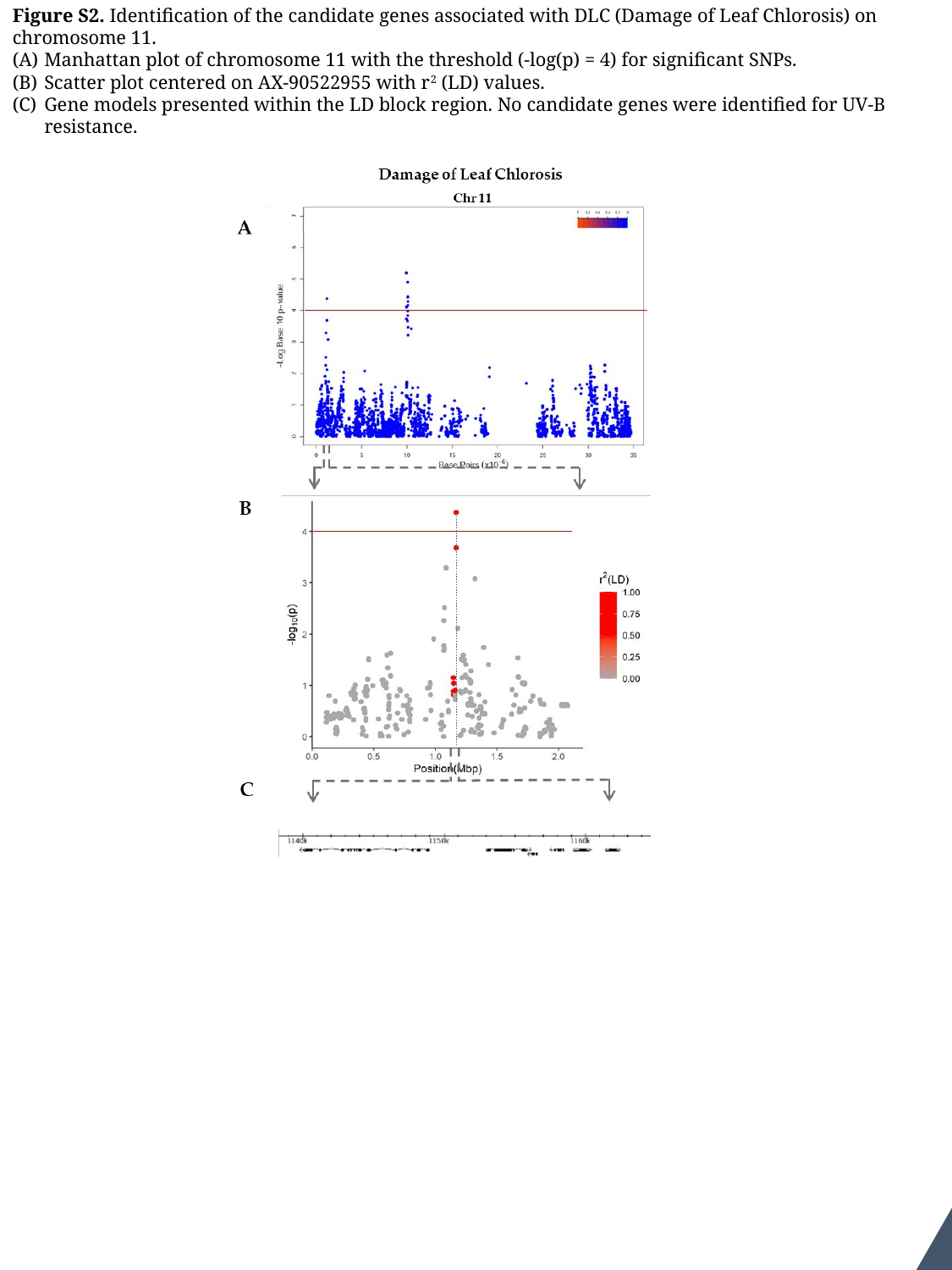

Figure S2. Identification of the candidate genes associated with DLC (Damage of Leaf Chlorosis) on chromosome 11.
Manhattan plot of chromosome 11 with the threshold (-log(p) = 4) for significant SNPs.
Scatter plot centered on AX-90522955 with r2 (LD) values.
Gene models presented within the LD block region. No candidate genes were identified for UV-B resistance.

## Slide 3
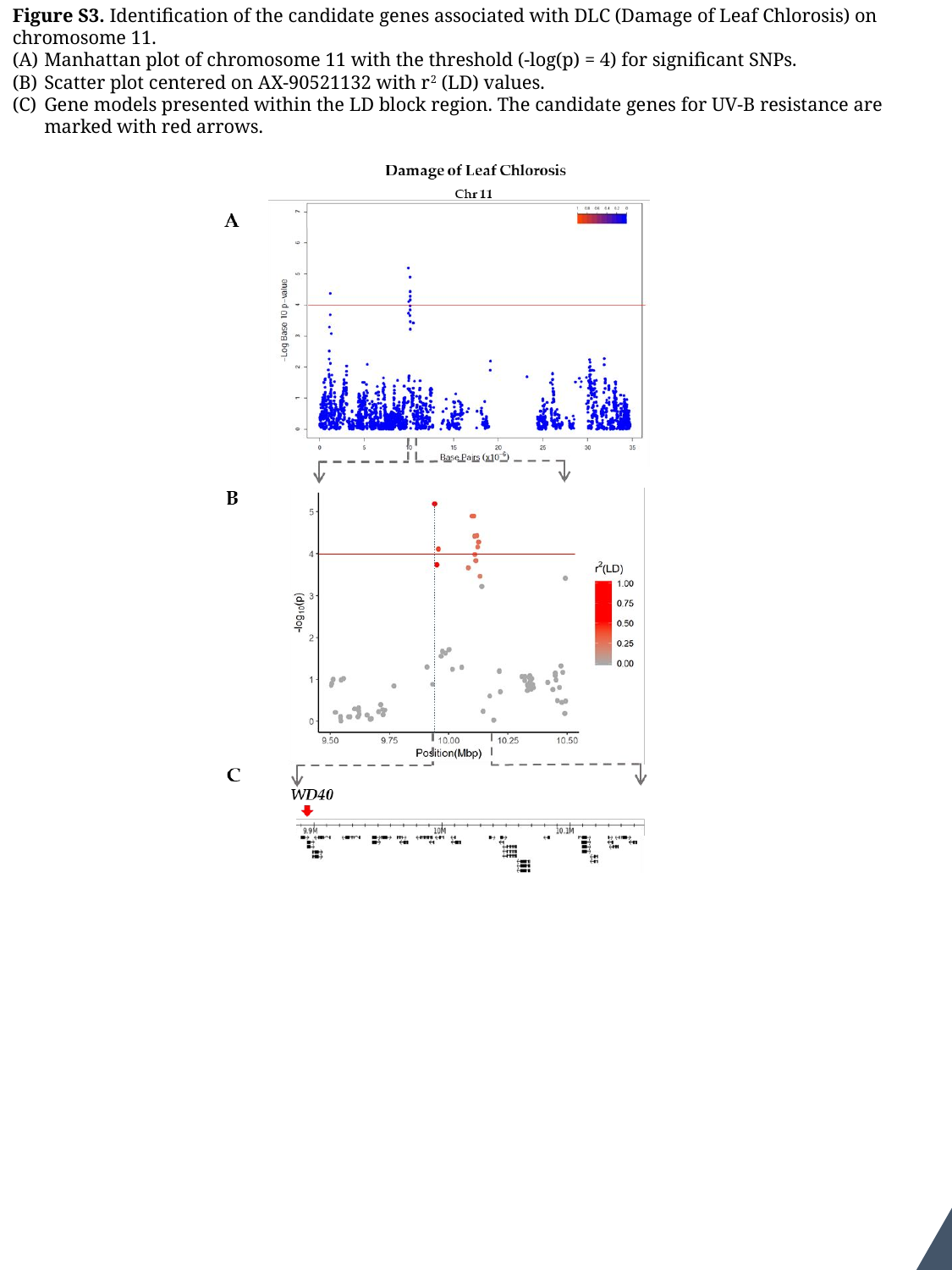

Figure S3. Identification of the candidate genes associated with DLC (Damage of Leaf Chlorosis) on chromosome 11.
Manhattan plot of chromosome 11 with the threshold (-log(p) = 4) for significant SNPs.
Scatter plot centered on AX-90521132 with r2 (LD) values.
Gene models presented within the LD block region. The candidate genes for UV-B resistance are marked with red arrows.
